# Transcriptome Analysis Reveals Genes Commonly Induced by *Botrytis cinerea* Infection, Cold, Drought and Oxidative Stresses in *Arabidopsis*

**DOI:** 10.1371/journal.pone.0113718

**Published:** 2014-11-25

**Authors:** Arjun Sham, Ahmed Al-Azzawi, Salma Al-Ameri, Bassam Al-Mahmoud, Falah Awwad, Ahmed Al-Rawashdeh, Rabah Iratni, Synan AbuQamar

**Affiliations:** 1 Department of Biology, United Arab Emirates University, Al-Ain, United Arab Emirates; 2 Department of Electrical Engineering, United Arab Emirates University, Al-Ain, United Arab Emirates; 3 Department of Mathematical Science, United Arab Emirates University, Al-Ain, United Arab Emirates; Institute for Sustainable Plant Protection, C.N.R., Italy

## Abstract

Signaling pathways controlling biotic and abiotic stress responses may interact synergistically or antagonistically. To identify the similarities and differences among responses to diverse stresses, we analyzed previously published microarray data on the transcriptomic responses of *Arabidopsis* to infection with *Botrytis cinerea* (a biotic stress), and to cold, drought, and oxidative stresses (abiotic stresses). Our analyses showed that at early stages after *B. cinerea* inoculation, 1498 genes were up-regulated (*B. cinerea* up-regulated genes; *BUG*s) and 1138 genes were down-regulated (*B. cinerea* down-regulated genes; *BDG*s). We showed a unique program of gene expression was activated in response each biotic and abiotic stress, but that some genes were similarly induced or repressed by all of the tested stresses. Of the identified *BUG*s, 25%, 6% and 12% were also induced by cold, drought and oxidative stress, respectively; whereas 33%, 7% and 5.5% of the *BDG*s were also down-regulated by the same abiotic stresses. Coexpression and protein-protein interaction network analyses revealed a dynamic range in the expression levels of genes encoding regulatory proteins. Analysis of gene expression in response to electrophilic oxylipins suggested that these compounds are involved in mediating responses to *B. cinerea* infection and abiotic stress through TGA transcription factors. Our results suggest an overlap among genes involved in the responses to biotic and abiotic stresses in *Arabidopsis*. Changes in the transcript levels of genes encoding components of the cyclopentenone signaling pathway in response to biotic and abiotic stresses suggest that the oxylipin signal transduction pathway plays a role in plant defense. Identifying genes that are commonly expressed in response to environmental stresses, and further analyzing the functions of their encoded products, will increase our understanding of the plant stress response. This information could identify targets for genetic modification to improve plant resistance to multiple stresses.

## Introduction

Plants are frequently exposed to environmental stresses that occur either simultaneously or in succession. Depending on the pathogen or the type of abiotic stress, plants attune their responses to activate resistance pathways [1]. In nature, plants exposed to abiotic stress may show enhanced resistance to pathogens, a phenomenon known as cross-tolerance [2]. This indicates that there is some crosstalk between signaling pathways mediating the responses to biotic and abiotic stress. Some studies have demonstrated that there are distinct pathways regulating plant responses to each individual stress, while others have shown that there is some coordination among plant responses to pathogens and abiotic stresses [3]–[6]. In general, different biotic and abiotic stress responses can be activated by unique or overlapping signaling pathways [6]–[8].

Many studies have focused on the plant response to individual stresses. The biotic stress response has been studied in the Arabidopsis-*Botrytis cinerea* pathosystem [4], [8]–[11]. *B. cinerea* is a necrotrophic pathogen that infects many plant species, including important crop species [12]. *Arabidopsis* plants infected with *B. cinerea* develop lesions, but do not mount a systemic acquired resistance response. Analyses of the *Arabidopsis* transcriptome or proteome during the defense response to *B. cinerea* infection have revealed up-regulation of genes encoding defense-related and regulatory proteins [5], [9], [13]–[15]. Similarly, there have been large-scale analyses of changes in the *Arabidopsis* transcriptome in response to cold, drought, or oxidative stresses [16]–[18].

The plant response to multiple environmental stresses differs from the response to an individual stress. Microarray analyses have revealed that plants exposed to combinations of biotic or abiotic stresses show a transcriptional response different from that induced by each individual stress [19]–[21]. For example, both tobacco (*Nicotonia attenuata*) and *Arabidopsis* showed different transcriptional responses to multiple insect herbivores than to a single pest [21], [22]. Therefore, Mittler and Blumwald proposed that a combination of stresses, rather than an individual stress, should be studied to understand the molecular mechanism of how plants sense, transduce, and adapt to multiple environmental conditions. Ultimately, this will allow us to develop crops tolerant to multiple stresses [23].

Plants exposed to a pathogen can become more susceptible to damage by subsequent abiotic stresses [24]. Similarly, long-term abiotic stress weakens plant defenses and increases susceptibility to pathogens [23]. A few studies have focused on the transcriptional regulation of responses to multiple biotic and abiotic stresses, and on the genes that are commonly induced by different stresses. A microarray analysis showed a distinct program of gene activation in response to simultaneous water deficit and nematode infection in *Arabidopsis* [25]. Moreover, combinations of flagellin (bacterial elicitor), cold, heat, high-light, and salt stress treatments caused transcriptomic changes that could not be predicted from the response to each individual stress treatment [26]. To date, there has been no report of a transcriptome analysis of plants simultaneously exposed to *B. cinerea* and abiotic stresses.

Genetic studies on *Arabidopsis* and tomato (*Solanum lycopersicum*) have shown that jasmonate (JA) and ethylene (ET) are key regulators of defense responses against necrotrophic infections [9], [27]–[29], while abscisic acid (ABA) regulates abiotic stress responses [3], [6]. Recently, two cyclopentenones, 12-oxo-phytodeniec acid (OPDA) and phytoprostanes (PP), were reported to accumulate after infection by various pathogens [4], [30]–[32] and in response to abiotic stresses [18], [33]. OPDA (the JA precursor) is produced enzymatically from α-linolenic acid and ultimately forms JA and/or its conjugates via the activity of OPDA reductase (OPR3) followed by three ß-oxidation steps [34]. Phytoprostane (PP) is produced nonenzymatically from α-linolenic acid via a free radical-catalyzed pathway. Mutations in *OPR3* and *expansin-like A2* (*EXLA2*) genes can modulate gene expression through cyclopentenone/COI1, independently of JA, under biotic stress [4], [35]. However, little is known about the role of electrophilic oxylipins OPDA or phytoprostane A_1_ (PPA_1_) in the plant response to *B. cinerea* infection.

Analyses of the molecular mechanisms involved in tolerance to pathogens and abiotic stress have generated large amounts of data. However, little is known about how individual biological processes function in the context of the entire cellular network. In the last decade, the integration of microarray data and coexpression network and protein–protein interaction (PPI) data has identified coregulated genes and/or protein complexes [36]–[38]. These studies, which aimed to identify differentially expressed genes and to determine their putative functions, have provided new insights into the basic mechanisms controlling cellular processes involved in tolerance to extreme conditions and pathogens *in planta*.

Studies on plant responses to individual stresses have revealed the genes and pathways that are activated during specific stress responses [39]. However, it is particularly useful to compare many different stress responses to identify the genes and pathways that are commonly induced by diverse stresses [20], [23]. This could reveal targets for genetic engineering to produce plants with tolerance to multiple stresses. In this study, therefore, we analyzed previously published datasets to identify stress-regulated genes involved in multiple stress responses, and to identify the components that regulate an overlap between biotic and abiotic stress responses. We performed a large-scale comparative transcriptomic analysis using publicly available microarray data. These data were obtained in studies on the transcriptomic response of *Arabidopsis* to *B. cinerea*, cold, drought, and oxidative stress. Our analyses revealed the genes expressed uniquely in response to each stress, and those expressed commonly in the responses to *B. cinerea* and other abiotic stresses. We identified the genes that were up- or down-regulated in all classes of stresses studied. A gene co-expression network analysis identified clusters of stress-responsive genes, which encoded regulatory proteins, in tightly co-expressed modules.

## Results

### Identification of differentially expressed genes in various stress responses

**Figure 1:**
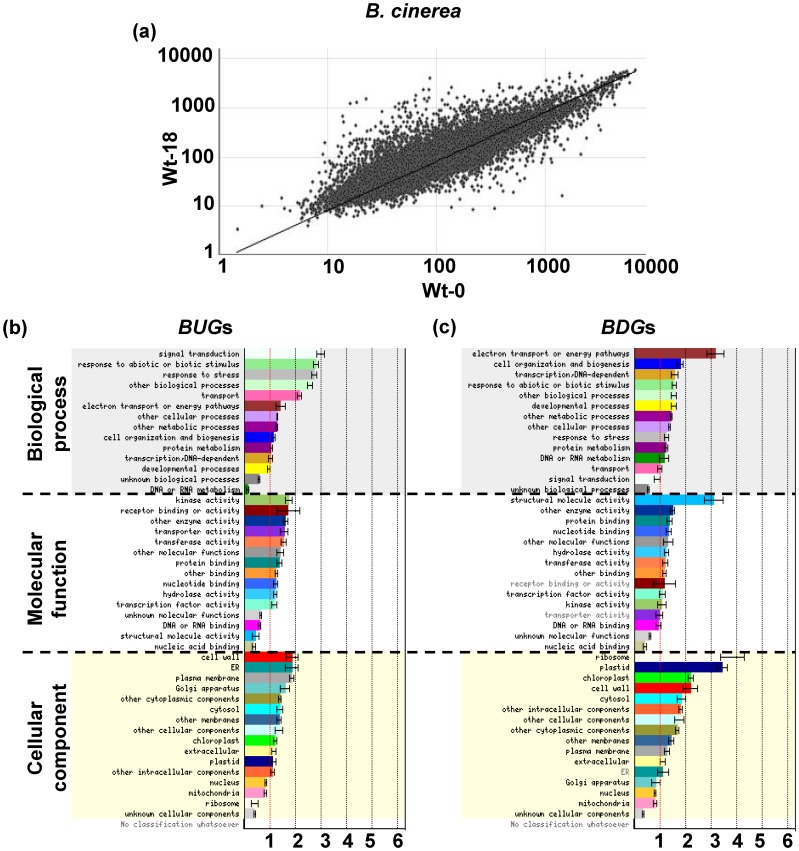
Scatter-plot comparisons of gene expression and functional classes of *BUG*s and *BDG*s. (A) Normalized expression value for each probe set in wild-type plants infected with *B. cinerea* at 18 hpi (Wt-18) is plotted on Y-axis; value in wild-type plants sampled before *B. cinerea* treatment (0 hpi; WT-0) is plotted on X-axis. (B) *BUG*s; and (C) *BDG*s at 18 hpi compared with 0 hpi in wild-type. Gene identifications for 1498 *BUG*s and 1138 *BDG*s were entered for this analysis. Error bars are SD. GO categories significantly over- or under-represented at *p*<0.05 are shown in black. Normalized frequency of genes to the number of genes on the microarray chip was determined as described elsewhere [72].

**Figure 2:**
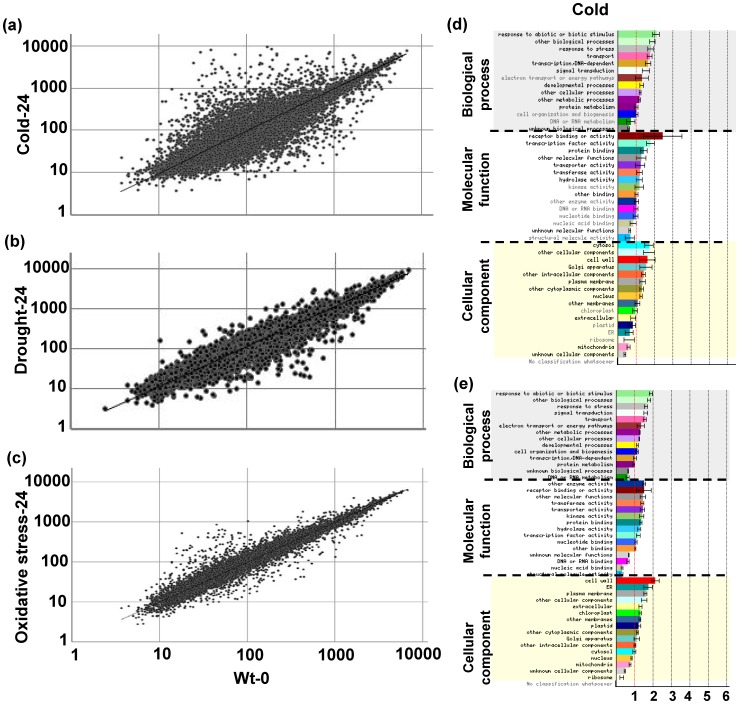
Scatter-plot comparisons of gene expression and functional classes of abiotic stress-regulated genes. Normalized expression value for each probe set in stressed plants with cold (A); drought (B); or oxidative stress (C) at 24 hpt is plotted on Y-axis; value in wild-type plants sampled before abiotic stress treatment (0 hpt; WT-0) is plotted on X-axis. (D) Cold-up-regulated genes; and (E) cold-down-regulated genes at 24 hpt compared with 0 hpt in wild-type. Gene identifications for 1248 cold-up-regulated and 1747 cold-down-regulated genes were entered for this analysis. Error bars are SD. GO categories significantly over- or under-represented at *p*<0.05 are shown in black. Normalized frequency of genes to number of genes on the microarray chip was determined as described elsewhere [72].

Previous studies on the gene expression profiles during the plant response to *B. cinerea* and other abiotic stresses focused on individual stresses [9], [40], [41]. In this study, we aimed to identify components of the regulatory networks involved in the response to *B. cinerea* infection and major abiotic stresses in *Arabidopsis*. A full microarray-based analysis of an *Arabidopsis* whole-genome Affymetrix gene chip (ATH1), representing approximately 25,000 genes, was downloaded from the NASC repository [40]. We analyzed this dataset to identify genes induced by *B. cinerea* infection and by abiotic stresses (cold, drought, and oxidative stress). First, we identified the differentially expressed genes by comparing the expression profiles between non-inoculated and *B. cinerea*-inoculated tissues (Figure 1A) and between non-treated or abiotic stress-treated wild-type plants (Figure 2A–C). For each gene, the fold change in expression was calculated by dividing the normalized gene expression level in the *B. cinerea*-infected or abiotic stressed wild-type sample by that in the corresponding control (no infection, no treatment).

We selected genes that were differentially expressed by at least two-fold at 18 hours post-inoculation (hpi) in *B. cinerea*-infected plants, or at 24 hours post-treatment (hpt) in wild-type plants subjected to abiotic stress (see Methods). Based on their transcriptional levels in the relevant tissues, *B. cinerea*-up-regulated genes (*BUG*s) and *B. cinerea*-down-regulated genes (*BDG*s) were identified. Overall, 1498 genes were up-regulated and 1138 genes were down-regulated in response to *B. cinerea* infection (Table S1). In total, 1248, 251, and 288 genes were up-regulated, and 1747, 302, and 247 were down-regulated in response to cold, drought, and oxidative stress, respectively (Table S2).

To validate the dataset and to better understand the regulation of gene expression during *B. cinerea* infection, we grouped *BUG*s or *BDG*s based on the functional similarity of their encoded products. The functional classification of *BUG*s and *BDG*s showed that signaling pathways, and cellular activities and components were associated with the response to this pathogen in *Arabidopsis*. AGI locus identifiers were categorized into 45 functional groups, and were then assigned into three main gene ontology (GO) categories: biological process, molecular function, and cellular component (Figure 1B,C). The dominant subcategory ‘signal transduction’ via plant hormones is a key component with plant defense against pathogens. For example, the effector genes plant defensin *PDF1.2* (*At5g44420*) and thionin *Thi2.1* (*At1g72260*) which have antimicrobial properties, were induced by ET/JA [9] and by *B. cinerea* (Table S1). Additionally, the ABA insensitive 1, *ABI1* (*At4g26080*), that is involved in ABA signal transduction, was upregulated by the same pathogen. This suggests that these plant hormones are tightly associated with defense against *B. cinerea*. The ‘kinase activity’ and ‘cell wall’ terms were also dominant subcategories in *BUG*s (Figure 1B). The cell wall-associated kinase, WAK1 (*At1g21250*), were also induced by *B.cinerea* (Table S1). There were also many genes in the ‘responses to abiotic and biotic stimulus’, ‘receptor activity’, and ‘endoplasmic reticulum’ subcategories (Figure 1B). The receptor-like kinase, RPK1 (*At1g69270*), which is a regulator of the ABA signal transduction pathway, was upregulated upon *B.cinerea* attack. The *BDG*s contained different dominant GO terms. For example, the major subcategories in the biological processes were associated with ‘electron transport or energy pathways’, and ‘cell organization and biogenesis’ (Figure 1C); and the dominant GO terms in the molecular functions were ‘structural molecule activity’ and ‘enzyme activity’. ‘Ribosome’ and ‘plastid’ were the dominant subcategories in the cellular component. This suggests a rapid repression of genes involved in plant metabolism upon inoculation with *B. cinerea*, consistent with previous findings [13]. Few of the *BUG*s and *BDG*s were in the ‘unknown biological processes’, ‘nucleic acid binding’, and ‘unknown cellular components’ subcategories (Figure 1B,C). The GO analysis indicated that many of the identified *BUG*s and *BDG*s were associated with biological processes and cellular components, respectively, upon *B. cinerea* attack. These findings are consistent with previous reports that *B. cinerea* induces/suppresses a number of genes encoding regulatory, developmental, organizational and structural proteins *in planta* [9], [10], [13], indicating potential connections between gene expression patterns and responses underlying plant resistance to *B. cinerea*.

Plants perceive cold, drought, and oxidative stress via cell membrane receptors. A signal is then initiated to activate cold-, drought- or oxidative stress-responsive genes and transcription factors that mediate stress tolerance [41]–[44]. We identified clear overlaps of the biological processes, molecular functions, and cellular components among the up-regulated or down-regulated genes in the responses to all three abiotic stresses (Figure 2D,E; Figure S1). The specificity of biotic and abiotic stress responses is controlled by a range of molecular mechanisms that may act together in a complex regulatory network. This suggests that there is common regulation of the responses to *B. cinerea* infection and abiotic stresses.

### Highly conserved expression status of genes common to *B. cinerea* and abiotic stress responses

**Figure 3:**
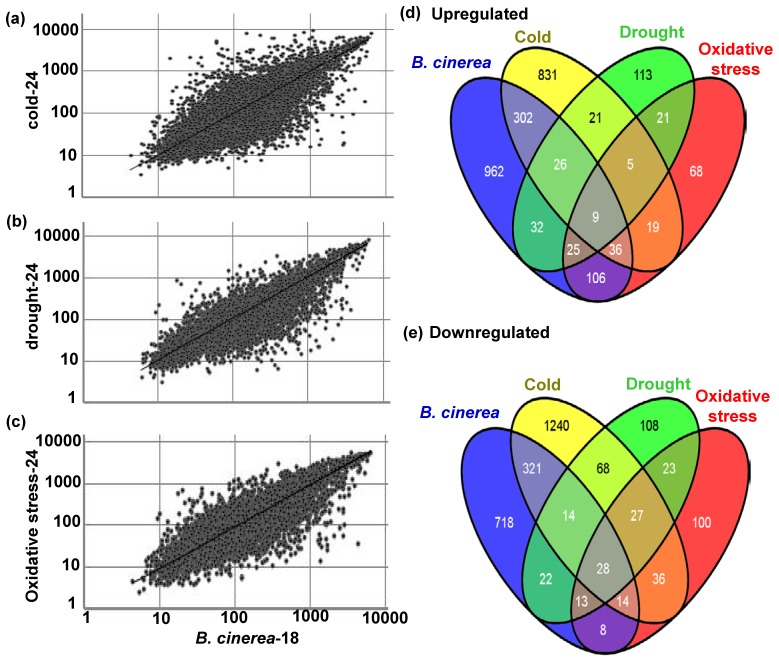
Scatter-plot comparisons of gene expression and number of *BUG*s and *BDG*s affected by abiotic stress. Normalized expression value for each probe set in wild-type plants infected with *B. cinerea* at 18 hpi (*B. cinerea*-18) is plotted on X-axis; value in stressed plants with cold (A); drought (B); or oxidative stress (C) at 24 hpt is plotted on Y-axis. Venn diagram showing the number of (D) *BUG*s and (E) *BDG*s at 18 hpi that are also affected by cold, drought, and oxidative stress at 24 hpt.

We compared the normalized transcript levels of all of the genes induced by *B. cinerea*- with their respective levels in plants subjected to abiotic stresses. We constructed scatter plots in which gene expression in response to *B. cinerea* was compared with that in response to drought, cold, or oxidative stress (Figure 3A–C). Direct comparison of gene expression levels after infection by *B. cinerea* at 18 hpi and abiotic stress (cold, drought or oxidative stress) at 24 hpt revealed remarkably similar expression patterns between these particular biotic and abiotic stresses. These results indicate that some genes may be involved in processes that are common among responses to different stresses.

We constructed a Venn diagram to illustrate which genes were induced by single stresses and which were induced by multiple stresses (Figure 3D,E). Specifically, we looked for relationships among sets of genes induced under diverse conditions. In looking at groups of genes induced under the four conditions, we detected large overlaps in gene expression among the biotic stress response (*B. cinerea*) and the abiotic stress response. For example, comparing *B. cinerea*-inoculated and cold-stressed plants, there were 373 commonly up-regulated genes, and 377 commonly down-regulated genes. Similarly, 92 genes were induced by *B. cinerea* infection and by drought treatment, and 77 were repressed in both of these treatments. Comparing *B. cinerea*-inoculated and oxidative stress-treated plants, there were 176 commonly up-regulated genes, and 63 commonly down-regulated genes. These results highlight overlaps in the responses to different stresses, and identify genes that showed up-regulation or down-regulation in all of the stress treatments (Table S3)

The datasets analyzed here were obtained from previous studies on seedlings subjected to four stresses; *B. cinerea*, cold, drought, and oxidative stress. Nine and 28 genes with increased and decreased expression levels, respectively, were shared among all four stress responses (Figure 3D,E). A detailed list of genes showing altered expressions in response to *B. cinerea*, cold, drought, and oxidative stress treatments is provided in Table 1.

**Table 1:** Changes in expression of up- or down-regulated genes during *B. cinerea* infection and abiotic stress treatments in Arabidopsis plants.

Gene ID	Gene family	Probe set	*B. cinerea*	Abiotic stress		
				Cold	Drought	Oxidative stress
*At1g73480*	Hydrolase	245734	2.37	15.39	2.07	2.33
*At4g34980*	Subtilisin-like serine protease 2 (SLP2)	253218	2.09	3.02	2.96	2.64
*At4g23600*	Coronatine induced 1 (CORI3)/Jasmonic acid responsive 2 (JR2)	254232	24.81	5.84	3.90	2.01
*At2g33380*	Responsive to desiccation 20 (RD20)	255795	5.15	13.81	5.24	3.30
*At3g05030*	Sodium proton exchanger 2 (NHX2)	259081	2.63	2.21	2.56	2.11
*At1g72380*	Unknown	260450	2.24	2.05	2.11	2.02
*At2g39420*	Esterase/lipase/thioesterase	266977	3.72	2.05	3.23	2.12
*At2g39250*	Schnarchzapfen (SNZ)	267010	2.41	4.98	2.02	2.37
*At2g41870*	Remorin	267538	2.54	3.35	3.20	2.45
*At5g64570*	Beta-xylosidase 4 (BXL4/XYL4)	247266	−2.35	−17.18	−3.23	−2.08
*At5g57560*	Touch 4 (TCH4)	247925	−2.63	−6.42	−7.02	−3.73
*At5g49450*	Basic leucine-zipper 1 (BZIP1)	248606	−2.94	−11.97	−2.80	−2.73
*At5g48430*	Aspartic-type endopeptidase/pepsin	248703	−2.08	−2.96	−2.12	−3.56
*At5g41080*	Glycerophosphoryl diester phosphodiesterase (GDPD2)	249337	−2.19	−14.76	−5.96	−5.14
*At5g24030*	SLAC1 homolog 3 (SLAH3)	249765	−2.65	−4.89	−2.86	−2.03
*At5g19120*	Aspartic-type endopeptidase/pepsin	249923	−2.08	−20.05	−3.17	−2.46
*At3g59900*	Unknown	251436	−2.88	−2.59	−6.24	−2.89
*At3g50560*	Short-chain dehydrogenase/reductase (SDR)	252167	−5.21	−4.99	−2.52	−2.58
*At3g50060*	MYB77	252193	−3.01	−5.28	−3.68	−2.14
*At3g48360*	BTB and TAZ domain protein 2 (BT2)	252367	−4.58	−3.51	−12.42	−4.07
*At4g37610*	BTB and TAZ domain protein 5 (BT5)	253061	−4.75	−18.55	−3.69	−3.24
*At4g21870*	26.5 kDa P-related heat shock (HSP26.5-P)	254384	−2.18	−12.29	−3.75	−2.75
*At4g12480*	pEARLI 1	254805	−8.34	−7.40	−21.24	−10.28
*At4g08950*	*Exordium* (EXO)	255064	−8.78	−18.67	−3.12	−2.11
*At4g02330*	PMEPCRB; pectinesterase	255524	−3.96	−2.10	−6.02	−4.98
*At4g01250*	WRKY22	255568	−2.15	−4.90	−4.45	−2.98
*At1g22190*	RAP2.4	255926	−3.84	−6.58	−3.00	−2.20
*At1g72060*	Serine-type endopeptidase inhibitor	256337	−4.22	−16.92	−4.37	−3.63
*At1g73830*	BR enhanced expression 3 (BEE3)	260070	−2.33	−8.34	−3.52	−3.39
*At2g43610*	Glycoside hydrolase family 19	260557	−2.38	−3.48	−2.56	−2.92
*At1g21910*	Dehydration response element-binding (DREB26)	260856	−5.69	−30.89	−14.22	−9.53
*At1g15550*	Gibberellin 3-oxidase 1 (GA3ox1; GA4)	261768	−2.86	−4.50	−2.47	−2.24
*At2g16586*	Unknown	263268	−2.20	−6.36	−2.94	−2.41
*At2g17880*	DNA J protein C24 (DJC24)	264788	−2.33	−2.10	−2.38	−3.00
*At1g24530*	Transducin/WD-40 repeat	265028	−4.69	−5.24	−6.87	−3.66
*At2g20670*	Unknown	265387	−4.33	−23.10	−3.75	−3.27
*At2g26980*	CBL-interacting protein kinase 3 (CIPK3)	266313	−3.18	−5.60	−4.01	−2.06

-Fold change in expression for each gene was calculated by dividing its expression level in *B. cinerea*- inoculated/abiotic-stressed sample by that in a non-inoculated/non-stressed sample (see Methods). A 2-fold change in expression represented up-regulated genes, and 0.5-fold change in expression represented down-regulated genes.

Enzymes (e.g., hydrolases, esterases), interacting kinases, and heat-shock proteins are known to regulate pathogen defense responses and abiotic stress tolerance. We found that *NHX2*, which encodes an Na^+^/H^+^ antiporter, was induced by all four stresses. *SLAH3* was repressed under all four stresses. These findings indicate that channels/transporters are involved in *stress* and *defense responses*. The up-regulation of *SNZ* and the down-regulation of *MYB77*, *WRKY22*, and *bZIP1* supported that transcription factors in the AP2 domain, MYB, WRKY, and BZIP families play important roles in mediating the responses to *B. cinerea* infection and abiotic stresses. Clearly, many different stresses regulate regulatory and structural genes involved in the plant defense response.

We selected the top-ranked commonly regulated genes in the responses to *B. cinerea*, cold, drought, and oxidative stress for coexpression and PPI network visualization analyses. Four commonly up-regulated and 12 commonly down-regulated genes were mapped to neighboring nodes and arranged according to their interactions (Figure S2). The input data for the PPI network included experimentally identified and computationally predicted interactions (Table S4). We avoided displaying coexpressed gene pairs with a low topological coefficient (TC). The TC is a relative measure of the extent to which a node shares neighbors with other nodes. This value was obtained using the Cytoscape plugin, Network Analyzer. In addition to the interactions between common up-regulated or down-regulated genes with the first neighboring genes, we showed the edges between interacting neighboring genes (Figure S2). The coexpression and PPI network analyses produced a large subset of 11713 nodes and 94048 edges (Table S4). Using this approach, we grouped genes into closely correlated modules based on their coexpression under various experimental conditions. The computed coexpression relationships between *B. cinerea* and abiotic stress-induced genes/nodes identified four genes: *NHX2*, *At2g39420* (esterase), *SLP2*, and *CORI3*. The whole genome clustering (grouping) revealed less complicated genetic network interactions than those of the repressed gene coexpression networks. Stress-related coexpression relationships reliably identified candidates that were robustly induced/repressed upon *B. cinerea* attack and abiotic stress treatments.

### Validation of expression profiles of common genes to *B. cinerea* infection

**Figure 4:**
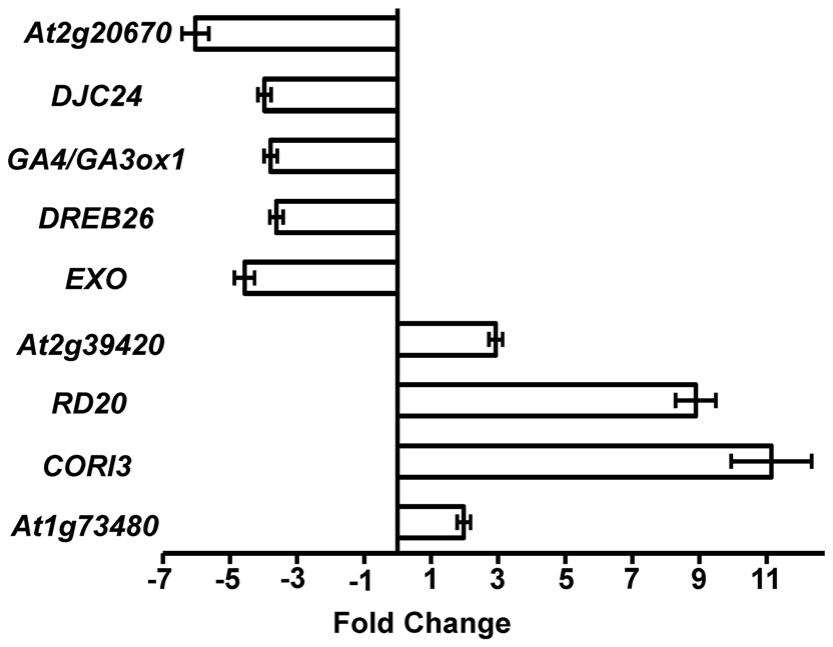
Expression of *B. cinerea*- and abiotic stress-regulated genes in response to *B. cinerea*. Relative expression levels obtained by qRT-PCR for selected common *B. cinerea*- and abiotic stress-up-regulated or -down-regulated genes obtained from Table (1) in response to *B. cinerea* infection at 18 hpi (see Methods). Expression of *B. cinerea*-inducible or -repressed genes was quantified relative to control conditions (no infection), and corrected for expression of control gene (*AtActin2*). Error bars for qRT-PCR values are standard deviations (*n*≥3).

To confirm the results of the previously published microarray analyses, we performed qRT-PCR on *Arabidopsis* leaves infected with *B. cinerea* at 18 hpi. We quantified the transcript levels of nine genes that showed changes in expression in response to the stress treatments, and compared the results with those obtained in microarray analyses (Figure 4). Although there were some differences between the qRT-PCR results and the microarray results in terms of the magnitude of fold changes, all of the tested genes (4 up-regulated; 5 down-regulated) showed similar trends in transcript accumulation in the qRT-PCR and microarray analyses. Therefore, the qRT-PCR results were consistent with the results from the microarray analysis.

### Regulation of cyclopentenone-induced genes during *B. cinerea* infection and abiotic stress

The cyclopentenone oxylipins, OPDA and PPA_1_, are formed via the enzymatic JA pathway and/or nonenzymatic free radical-catalyzed pathway, respectively [45], [46]. We searched the *B. cinerea*-regulated genes (Table S1) to identify genes responsive to OPDA and/or PPA_1_ by comparing *BUG*s and *BDG*s with genes reported to be induced in OPDA- and/or PPA_1_-treated *Arabidopsis* plants. Table 2 shows genes induced by OPDA treatment [47] and by *B. cinerea* attack; these genes were designated as OPDA/*B. cinerea*-up-regulated genes (*OBUG*s). The identified *OBUG*s were induced more than two-fold by both OPDA treatment and *B. cinerea* infection. Of the OPDA-up-regulated genes identified [47], approximately 61% (45/74) were also up-regulated by *B. cinerea* infection (Table 2). The *OBUG*s encoded a subset of proteins including transporters, zinc-finger, UDP-glycosyltransferase, heat shock, ABA-responsive proteins, and other related proteins. None of the OPDA-down-regulated genes were repressed by *B. cinerea* infection. The previously identified abiotic stress-responsive genes (Table S2) were further analyzed to determine which ones were induced by OPDA treatment and which were induced by infection with *B. cinerea*. Two-fold induction was set as the threshold value for induction. Of the 45 *OBUG*s identified above, 9 (20%) were also induced by cold stress, and 17(37.8%) were also induced by oxidative stress (Table 2). Three of the OPDA-down-regulated genes were repressed by cold, drought, or oxidative stress (Table S5).

**Table 2:** Genes up-regulated by PPA_1_, OPDA, *B. cinerea* inoculation and abiotic stresses and dependent on *TGA2/5/6*.

Array Element	Gene Locus	Description	Normalized Fold Induction^a^				
			PPA_1_ b	OPDA^b^	TGACG^b^	*B. cinerea* c	Abiotic stress^d^
***OBUG*** **s**							
*249417_at*	*At5g39670*	Calcium-binding EF-hand family protein	N	2.8		2.2	
*250781_at*	*At5g05410*	Dehydration-responsive element-binding (DREB2A)	N	4.4		3.4	C,Ox
*256576_at*	*At3g28210*	Zinc-finger protein (PMZ)	N	17.4		7.9	C,Ox
*247655_at*	*At5g59820*	Zinc-finger protein (ZAT12/RHL41)	N	3.5		3.6	C,Ox
*264968_at*	*At1g67360*	Rubber elongation factor (REF)	N	2.0		3.5	C
*251336_at*	*At3g61190*	BON1-associated protein 1 (BAP1)	N	2.5		2.6	C
*265499_at*	*At2g15480*	UDP-glucose transferase (UGT73B5)	N	6.7		3.1	Ox
*252515_at*	*At3g46230*	Heat-shock protein 17.4 (HSP17.4)	N	12.4		3.3	Ox
*254890_at*	*At4g11600*	Glutathione peroxidase 6 (GPX6)	N	3.2		5.2	C
*249719_at*	*At5g35735*	Auxin-induced protein	N	3.4		12.3	C,Ox
*264929_at*	*At1g60730*	Aldo/keto reductase (NADP activity)	N	4.6		5.4	Ox
***PBUG*** **s**							
*262517_at*	*At1g17180*	GSTU25	17	N		10.8	Ox
*266267_at*	*At2g29460*	GSTU4/GST22	3.7	N		9.3	Ox
*266752_at*	*At2g47000*	Multidrug-resistant ABC transporter (MDR4)	8.7	N		6.6	Ox
*256221_at*	*At1g56300*	DNAJ heat shock	3.5	N		26.7	C
*252984_at*	*At4g37990*	Cinnamyl-alcohol dehydrogenase (CADB2)/Elicitor activated gene (ELI3-2)	15	N		75.2	Ox
***PBDG*** **s**							
*256275_at*	*At3g12110*	ACT11	−3.6	N		−4.2	C
***OBUG*** **s and ** ***PBUG*** **s**							
*261763_at*	*At1g15520*	ABC transporter (PDR12)	24.5	18.7	P	22.6	Ox
*258277_at*	*At3g26830*	Phytoalexin deficient 3 (PAD3)	9.6	7.9		18.3	Ox
*249942_at*	*At5g22300*	Nitrilase 4 (NIT4)	9.3	6.6	P	4.1	
*266995_at*	*At2g34500*	Cytochrome P450 family (CYP710A1)	5.8	3.8	P	9.3	Ox
*250983_at*	*At5g02780*	Glutathione transferase lambda 1 (GSTL1); ln2-1	5.2	3	P	5.4	
*258921_at*	*At3g10500*	NAC domain containing protein 53 (ANAC053)	4.7	2.1	P	3.1	
*267168_at*	*At2g37770*	Aldo/keto reductase (AKR4C9)	4.4	3.7	P	7.9	
*250948_at*	*At5g03490*	UDP-glucoronosyl/UDP-glucosyl transferase	3.7	2.5	P	2.4	D,Ox
*251176_at*	*At3g63380*	Calcium-transporting ATPase (ACA12)	3.5	5.9	P	20.4	Ox
*258957_at*	*At3g01420*	Alpha-dioxygenase 1 (ALPHA-DOX1)	3.4	2.1	P	27.9	
*259911_at*	*At1g72680*	Cinnamyl alcohol dehydrogenase (CAD1)	3.3	2	P	2.9	
*262381_at*	*At1g72900*	Disease resistance protein (TIR-NBS class)	3.3	3.7	P	4.1	Ox
*262607_at*	*At1g13990*	Expressed protein	3	3	P	4.1	
*246042_at*	*At5g19440*	Alcohol dehydrogenase	2.9	2.4		3.2	
*261957_at*	*At1g64660*	methionine gamma-lyase (MGL)	2.8	6.5		3.9	
*257951_at*	*At3g21700*	GTP binding (SGP2)	2.7	2.3		4.7	Ox
*249860_at*	*At5g22860*	Ser carboxypeptidase S28 family	2.7	3.4	P	6.5	Ox
*263517_at*	*At2g21620*	Responsive to desiccation 2 (RD2)	2.7	2.1	P	5.5	C,Ox
*262482_at*	*At1g17020*	Senescence-related gene 1 (SRG1)	2.4	2.6		52.7	
*250054_at*	*At5g17860*	Calcium exchanger 7 (CAX7)	2.3	3.9		2.3	
*260551_at*	*At2g43510*	Trypsin inhibitor protein (TI1)	2.3	7.3		4.6	
*245768_at*	*At1g33590*	Disease resistance LRR protein-related	2.3	2.5	P	3.3	
*266000_at*	*At2g24180*	Cytochrome P450 monooxygenase (CYP71B6)	2.1	2		2.9	
*247177_at*	*At5g65300*	Expressed protein	2.2	2.5	P	5.0	C,Ox

a Normalized fold induction = normalized OPDA/PPA_1_ treatment, *B. cinerea* inoculation or abiotic stress/normalized no OPDA/PPA_1_ treatment, no *B. cinerea* inoculation or no abiotic stress.

b Normalized-fold induction of genes by PPA_1_ and/or OPDA (75 µM).

Threshold value for TGA2/5/6-dependent up-regulation was two-fold in *Arabidopsis* wild-type plants relative to controls but no induction in *tga2/5/6*. OPDA-up-regulated genes data were obtained from [47] at 3 hpt. PPA_1_-up-regulated genes data were obtained from [32] at 4 hpt. PPA_1_- and OPDA-induced genes data were obtained from [32] at 4 hpt.

c Normalized fold induction of genes by *B. cinerea*.

Threshold value for up-regulation was at least twofold in *Arabidopsis* wild-type plants relative to controls. *B. cinerea*-induced genes data were obtained at 18 hpi [40] (Table S1).

d Normalized fold induction of genes by cold, drought, or oxidative stresses.

Threshold value for up-regulation was at least twofold in *Arabidopsis* wild-type plants relative to controls. Abiotic stress-induced genes data were obtained at 24 hpi [40] (Table S2).

N, not expressed; +, P, Present; −, downregulation.

We also compared the *B. cinerea*-regulated genes with PPA_1_-responsive genes [32]; this group was designated as PPA_1_/*B. cinerea*-up-regulated genes (*PBUG*s). As described above, two-fold induction was set as the threshold value for up-regulation. Of the 73 genes induced by PPA_1_ [32], 29 (39.7%) were also induced by *B. cinerea* (Tables 2). An analysis of the functions of the genes induced by PPA_1_/*B. cinerea* showed that *PBUG*s encoded proteins related to detoxification or to stress responses. These proteins included cytochrome P450, glutathione S-transferases, ABC transporters, and heat shock factors/proteins. Only three *PBUG*s (*At1g56300*, *At2g21620* and *At5g65300*) were induced by cold (Table 2). Our analyses indicate that most of these genes are transcriptionally regulated during the plant response to PPA_1_, *B. cinerea*, and oxidative stress. Surprisingly, the only *PBUG* (*At5g03490*) which was also induced by drought stress, encodes an UDP-glucoronosyl/UDP-glucosyl transferase enzyme. One gene, *Act11* (*At3g12110*), was repressed by PPA_1_ treatment and by *B. cinerea* infection, was also down-regulated by cold. Regardless of the regulation by *B. cinerea* infection, the list of genes that were induced/repressed by OPDA and/or PPA_1_ and by cold, drought or oxidative stress was shown in Table S5. Together, the results of these analyses suggest that *B. cinerea* and oxidative stress responses are mediated by the non-enzymatic oxylipin-dependent pathway.

### Regulation of *OBUG*s and *PBUG*s by TGA transcription factors

Cyclopentenones may function independently from JA [32], [48]. Many genes containing a TGA-motif (TGACG) in the 500 bp upstream of their promoters contain binding sites for TGA transcription factors [49]. We determined whether genes commonly induced in the response to *B. cinerea* and to PPA_1_ and OPDA were regulated by TGA transcription factors by analyzing their expression levels in a *tga2/5/6* mutant. For this analysis, we used data reported in [32]. We set our analysis at two-fold up-regulation for the induction by PPA_1_ and OPDA treatments, *B. cinerea* infection, and abiotic stress. Of the 27 genes up-regulated by PPA_1_ and OPDA that were dependent on the presence of *TGA2/5/6* [32], 16 (59.3%) were also induced by *B. cinerea* (Table 2). Of these *OBUG*s/*PBUG*s that were TGA-dependent, 8 were also induced by oxidative stress; very few genes were also induced by cold or drought. Thus, in *Arabidopsis*, *B. cinerea* induces many genes that are also induced by treatments with PPA_1_ and OPDA. Together, these data suggest that there is a common pathway, which involves TGA transcription factors, involved in the *B. cinerea* and oxidative stress responses.

### Validation of cyclopentenone-inducted genes by *B. cinerea*

**Figure 5:**
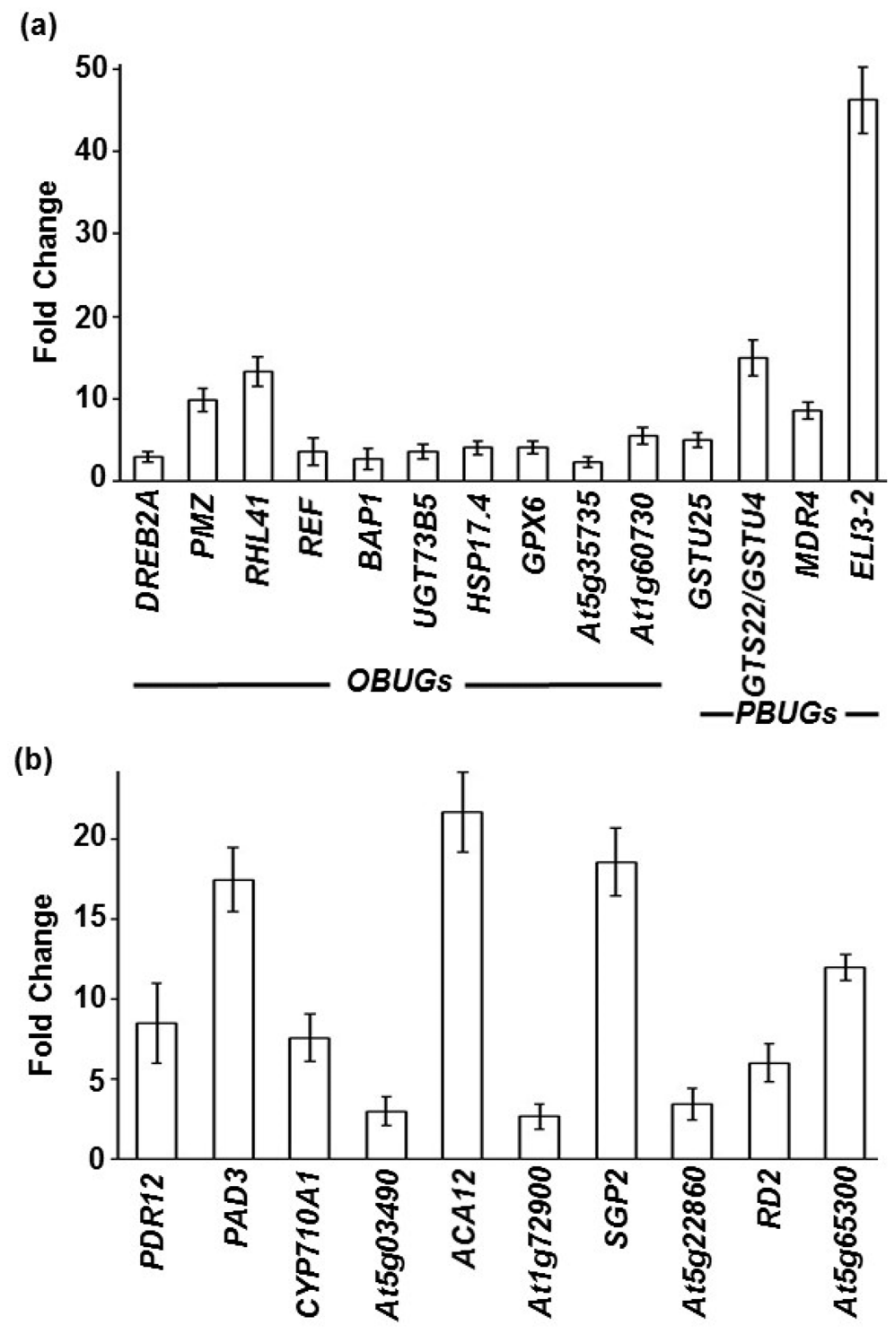
Expression of *OBUG*s/*PBUG*s and abiotic stress-regulated genes to *B. cinerea* infection. Relative expression levels obtained by qRT-PCR for common (A) *OBUG*s or *PBUG*s and abiotic stress-up-regulated genes; and (B) *OBUG*s/*PBUG*s and abiotic stress-up-regulated genes after infection with *B. cinerea* at 18 hpi (see Methods). Gene expression of *OBUG*s or *PBUG*s was normalized relative to control conditions (no infection), and corrected for expression of control gene (*AtActin2*). Error bars for qRT-PCR values are standard deviations (*n*≥3). Data shown in (A) and (B) were obtained from Table 2.

Next, we verified the microarray data and compared the genes induced by *B. cinerea*, abiotic stresses, and OPDA and/or PPA_1_ [32], [47]. We evaluated changes in gene transcript levels in response to *B. cinerea* infection by qRT-PCR analysis (Figure 5). We analyzed the transcript levels of genes encoding zinc finger transcription factor DNA-binding proteins. *PMZ* and *RHL41* were rapidly induced by OPDA (Table 2) and were up-regulated by *B. cinerea* (Figure 5A). *DREB2A*, which encodes a DREB subfamily A-2 protein (an ERF/AP2 transcription factor), was induced by cold stress [50] and by *B. cinerea*. Upon *B. cinerea* infection, three *OBUG*s (*UGT73B5*, *HSP17.4* and *GPX6*) were up-regulated, as demonstrated by the qRT-PCR results (Figure 5A) and the microarray data (Table 2). The induction of *GSTU4*, *GSTU25*, *MDR4*, and *ELI3-2* by *B. cinerea* suggests that these regulators play a role in stress responses. Expression of the detoxifying gene *PDR12* (ABC transporter) was also induced by *B. cinerea*. Except for *SGP2*, all of the other *OBUG*s or *PBUG*s analyzed showed similar patterns of expression in both the microarray datasets (Table 2) and the qRT-PCR analyses (Figure 5B). Our analyses suggest that oxylipins modulate gene expression in response to *B. cinerea* infection, and that these responsive genes are differentially regulated depending on the stress.

## Discussion

There have been many studies on large-scale transcriptomic changes in response to the necrotrophic fungal pathogen *B. cinerea* [5], [9], [13]–[15] and abiotic stresses [16]–[18]. Here, we investigated in detail the *Arabidopsis* response to *B. cinerea* infection and environmental stresses by analyzing previously published datasets. These datasets represented the transcriptomic differences between *Arabidopsis* leaves inoculated/treated with *B. cinerea*/abiotic stress (cold, drought, or oxidative stress) and non-inoculated/non-treated leaves. We initially assured that the transcript responses we detected to the four single stresses were comparable to those described by others. This “greenlight” permitted us to further analyze the transcript profiles responding to these stresses. Thus, we record a couple of potential limitations that are associated with the stress applications in this research as well as other studies. First, we analyzed transcriptome data of shoot tissues only after individual stress treatments at a single time point based on previous studies. As a result, we were not able to detect the temporal pattern of plant responses to single stresses. In our attempts to detect plant responses caused specifically by the environmental stress and to eliminate any indirect consequences of the particular stress, we chose a sampling time point prior to the appearance of visible stress symptoms. Second, we did not determine the relative intensities of the individual stresses assessed. Regardless of these caveats, we anticipate that our transcriptome data analyses can be a valuable source for researchers to understand the complex regulatory pathways and to further identify genes linked to environmental insult.

We identified that 1498 (6.6% of the transcriptome) and 1138 (5%) genes were up-regulated (*BUG*s) and down-regulated (*BDG*s), respectively, by *B. cinerea* infection at 18 hpi. We selected 18 hpi as the best time point to compare differences in gene expression, because it was reported that most changes in gene expression occur between 18 and 30 hpi [9], [13]. According to the GO classifications shown in Figure 1, the *BUG*s and *BDG*s encode proteins related to plant responses to stimuli and stresses, transport and energy pathways, and other cellular, metabolic, and biological processes. This result confirms that the *BUG*s and *BDG*s encode proteins with roles in signal transduction pathways and resistance to *B. cinerea* [9], [13], [14]. The different expression levels of *BUG*s and *BDG*s in different subcellular locations in the cytosol and the cell wall is consistent with the role of extracellular and intracellular components in activating gene expression in the response to *B. cinerea* attack.

We identified 1248 (5.5%), 251 (1.1%), and 288 (1.3%) up-regulated genes and 1747 (7.7%), 302 (1.3%), and 247 (1.1%) down-regulated genes in response to cold, drought, and oxidative stresses, respectively, at 24 hpt. These findings suggest that a unique program of gene expression is activated in response to *B. cinerea* or abiotic stress. We also compared the genes induced by *B. cinerea* and the various abiotic stresses to determine which were specific to each stress response, and which were common among the stress responses. Approximately 25%, 6%, or 12% of the 1498 *BUG*s were also induced by cold, drought, or oxidative stress, respectively. About 33%, 7%, or 5.5% of the 1138 *BDG*s were repressed by cold, drought, or oxidative stress, respectively. In general, gray mold, the disease caused by *B. cinerea*, occurs under diverse production conditions, even at 0–10°C storage, and causes significant yield losses. The *EXLA2* transcript levels decreased when *Arabidopsis* plants were exposed to *B. cinerea* infection, but increased in response to cold and salt treatments [4]. In a previous study, the *B. cinerea*-susceptible mutant *bos1* showed impaired tolerance to drought, salinity, and oxidative stress; the tolerance to these stresses was shown to be mediated by the reactive oxygen intermediates generated in the plant response [10]. The impaired tolerance of the *bos1* mutant to *B. cinerea* and abiotic stresses can be attributed to the shared responsive genes among *B. cinerea* and abiotic stress responses. Among all of the *BUG*s, nine were induced by all of the tested stresses (Figure 3D). Among all of the *BDG*s, 28 were repressed by all of the tested stresses (Figure 3E). Similar analyses of biotic and abiotic stress responses in rice (*Oryza sativa*) [37] have identified a similar set of commonly up-regulated and down-regulated genes to those identified in *Arabidopsis*.

Plant hormones play central roles in multi-environmental stress responses. Depending on the nature of the pathogen, induced resistance responses are mediated by various phytohormones, including salicylic acid (SA), JA, ET, and ABA [51]–[53]. While several studies have suggested that biotrophic pathogens commonly activate the SA-dependent defense response, others showed a limited role of SA and SA-dependent defense responses against *B. cinerea* in *Arabidopsis* [10], [11]. Necrotrophic pathogens, including *B. cinerea*, activate JA/ET-dependent signaling pathways [52]. ABA is a major regulator of the plant response to abiotic stress, and it also regulates disease resistance [54]–[57]. Together, SA, ET/JA, and ABA act together or antagonistically to regulate plant responses to pathogens and abiotic stress factors [53], [58]. One of the commonly induced genes was *CORI3/JR2*, which encodes cystine lyase, an enzyme that generates an ET precursor. In another study, *COR13*/*JR2* transcript levels were elevated in response to the hemibiotrophic pathogen *Pseudomonas syringae*, wounding, and JA [59]–[61]. In *Arabidopsis*, the ABA-induced gene *RD20*, which encodes a member of caleosin family, is also induced by drought and *B. cinerea* [62]. The microarray data and our qRT-PCR analyses demonstrated that *CORI3* and *RD20* were induced by *B. cinerea* attack and by cold, drought, and oxidative stresses. Three of the *BDG*s were *GDPD2*, *HSP26.5-P* and *At2g20670*, consistent with the results of a previous study on *B. cinerea* [13]. These three *BDG*s were also down-regulated by cold, drought, and oxidative stress. Our analyses suggest that each individual stress treatment induces a unique set of differentially expressed genes, but that a subset of nine genes is induced in response to *B. cinerea* and cold, drought, and oxidative stress. However, the thresholds selected to represent induction (2-fold) or repression (0.5-fold) of gene expression were high; therefore, there may be more genes that are commonly induced by several stresses than were detected in this study.

We conducted coexpression and PPI network analyses using Cytoscape software to identify genes involved in the defense response to *B. cinerea* infection and abiotic stresses. This analysis aimed to identify potential key regulators of the defense response and to predict regulatory interactions/relationships. As well as showing the novelty of each response, the analysis allowed us to visualize the PPI network and multiple dynamic gene coexpression networks to further understand plant responses to multiple stresses. Overall, the microarray and coexpression network analyses indicate that there is a complex response to multiple stresses. This response involves overlapping among different pathways and the synergistic and antagonistic regulation of biotic and abiotic stress response pathways.

We examined whether the genes up-regulated by PPA_1_ and/or OPDA [32], [47] also showed changes in expression in response to *B. cinerea* and abiotic stresses. Electrophilic oxylipins accumulate in plants during pathogen infection (including *B. cinerea*) and abiotic stress [30], [31]. It was reported that 38% of the genes in *Arabidopsis* are induced by PPA_1_ and *B. cinerea* [32]. Analyses of the microarray data showed that ∼61% and ∼40% of the genes induced by OPDA and PPA_1_ were also up-regulated by *B. cinerea*, respectively. Among the other genes that responded to PPA_1_ or OPDA [32], [47], *PMZ* and *RHL41* were also induced by *B. cinerea* (Figure 5). This suggests that there is a common regulation between electrophilic oxylipins and *B. cinerea*. Because electrophilic oxylipins accumulate in plants during pathogen infection (including *B. cinerea*) and abiotic stress [30], [31], we hypothesized that cyclopentenone levels and abiotic stress are also co-regulated in *Arabidopsis*. To test this hypothesis, we extended our analyses to determine whether *OBUG*s or *PBUG*s were also induced by cold, drought and oxidative stress (Table 2). Strikingly, most of the *OBUG*s and *PBUG*s were induced by oxidative stress. These results suggest that cyclopentenone levels and the abiotic stress response are co-regulated *in planta*, consistent with the results of other reports [63], [64].

Next, we determined whether the regulation of *OBUG*s and *PBUG*s was dependent on *TGA2*, *TGA5*, and *TGA6*. Eventhough we found a number of cyclopentenone-induced genes which were also induced by *B. cinerea* infection; about 59% of these *OBUG*s/*PBUG*s were dependent on TGA transcription factors, a result that was also validated by qRT-PCR. Interestingly, 33% of the TGA-dependent *OBUG*s and *PBUG*s were induced by oxidative stress. A recent study on the *exla2* mutant illustrated an overlap among its responses to *B. cinerea*, oxidative stress, and PPA_1_, but not JA [4]. Our results are consistent with a previous report that the transcript levels of *CYP710A1* and *ACA12* were strongly increased by *B. cinerea* infection [65], [66], possibly in a TGA-dependent manner. More research is required to test this hypothesis.

Our analyses suggest that there is common regulation of gene expression in the responses to electrophilic oxylipins, *B. cinerea*, and oxidative stress. This study has also identified potentially new candidate genes functioning in plant defense. Reverse genetic screening using mutant lines with deletions and/or overexpressions of the putative coexpressed genes (identified from coexpression networks) will help to discover new genes that function in the defense response *in planta*. Transcriptome analyses can highlight which genes show differential expression under certain conditions. However, changes in gene expression do not necessarily mean that there will be changes in the abundance or activity of their encoded products. Therefore, in future research, it will be important to evaluate the similarities and differences in the proteome and in the activities of various proteins among different stress responses. Identifying key regulators of the crosstalk between biotic and abiotic stress signaling pathways is a basic prerequisite for developing crop plants tolerant to multiple stresses.

## Conclusions

The results of these analyses suggest that there is overlapping among genes or pathways involved in the responses to biotic stresses and to abiotic stresses in *Arabidopsis*. Changes in the transcript levels of genes encoding components of the cyclopentenone signaling pathway in response to biotic and abiotic stresses suggest that the oxylipin signal transduction pathway plays a role in plant defense. Identifying genes that are commonly expressed in response to multiple stresses, and analyzing the functions of their encoded products, will increase our understanding of the plant stress response. This information could identify targets for genetic modification to improve plant resistance to multiple stresses.

## Materials and Methods

### Data source and analysis

Datasets were not subjected to any additional normalization, as all had been normalized when we obtained them. We downloaded “signal” data from NASCArrays [affy.arabidopsis.info/link_to_iplant.shtml] [40] for each stress; where only the “shoots” class was analyzed. The reference numbers are as follows: control, NASCArrays-137; cold stress, NASCArrays-138; drought stress, NASCArrays- 141; oxidative stress, NASCArrays-143; and *B. cinerea*, NASCAarrays-167. Log_2_-transformed expression level data were used to generate scatter plots to detect the effect of *B. cinerea* infection at 18 hpi or specific abiotic stress treatment at 24 hpt on gene expression. Three replicates from 80 biologically different samples were compared. There were 22810 genes in each sample. In all samples, probes having negative or zero expression signal values were removed. At the tested time point, the overall difference in gene expression between non-treated/non-inoculated (control) and treated/inoculated samples was determined by pairwise comparison. The normalized-fold change value for each gene was calculated by dividing the expression level in a treated/inoculated sample by the expression level in a non-treated/non-inoculated sample. A two-fold or half-fold (unless otherwise stated) difference in expression level between treated/inoculated and non-treated/non-inoculated samples at *P*≤0.05 was set as the threshold for considering a gene be up-regulated or down-regulated, respectively. The cutoffs of the fold change and p-value were chosen to filter false positives and to compare our data analyses with those in the microarray literatures. The identities of genes across microarray datasets were established using The Arabidopsis Information Resources (TAIR; www.arabidopsis.org). We used microarray data from seedlings treated with OPDA and PPA_1_ obtained in previous studies [32], [47].

### *In vitro* assays for cold, drought, and oxidative stress

We analyzed data from an original study on the responses of Arabidopsis to various stress conditions [40]. In that study, the experiments were conducted as described in the following paragraphs.

Seeds were surface-sterilized in 70% ethanol for 2 min, then in 30% Clorox solution containing 0.01% Tween for 10 min. The seeds were rinsed five times in sterile water and then sown on medium containing Murashige and Skoog (MS) salts, 2% sucrose, and 0.7% (w/v) purified agar, unless otherwise stated. Plates were kept at 4°C for 48 h to synchronize germination, transferred to growth chambers with fluorescent lights, and maintained under the environmental conditions as described in [41] with some modifications.

Stress treatments were applied in *in vitro* conditions using 11-day old seedlings as the plant material. For drought stress, seedlings were kept under a dry air stream (clean bench) for 24 h, until 10% of the fresh weight had been lost. For the cold-stress treatment, seedlings were placed on ice to cool rapidly and then kept at 4°C for 24 h in a cold room. For the oxidative stress treatment, seedlings were exposed to 10 µM paraquat (methyl viologen) for 24 h. For the control, the seedlings were treated with liquid-MS medium (control). All treatments and preparations were conducted using the same batch of seedlings, as described in [40].

### Plant growth, pathogen culture, and disease assay

We analyzed data from an original study on *Arabidopsis* plants (ecotype Col-0) infected with *B. cinerea* [40]. In that study, the experimental conditions were as follows: *Arabidopsis* leaves were inoculated by placing four 5-µl drops of a 5×10^5^ spore solution onto each leaf. Control leaves were spotted with droplets of potato dextrose broth medium (24 g L^−1^). The responses to *B. cinerea* infection were assayed at 18 and 48 hpi of adult leaves.

For the qRT-PCR analysis, the *B. cinerea* strain *BO5-10* was grown on 2×V8 agar (36% V8 juice, 0.2% CaCO3, 2% Bacto-agar). To initiate and maintain fungal cultures, pieces of agar containing mycelium were transferred to fresh 2×V8 agar and incubated at 20–25°C. Conidia were collected from 10-day-old cultures as described in [9]. Five weeks old plants grown in soil were spray-inoculated with 3×10^5^ spores mL^−1^ *B. cinerea* spore suspensions, using a Preval sprayer (Valve Corp., Yonkers, NY, USA). The control plants were sprayed with 1% Sabouraud maltose broth buffer. To establish disease, plants were kept under a sealed transparent cover to maintain high humidity in a growth chamber under the following conditions: 21°C day/18°C night temperature, 12-h light/12-h dark photoperiod.

### RNA extraction and expression analysis

RNA extraction and qRT-PCR expression analyses were performed as described previously [4]. qRT-PCR was performed using gene-specific primers, with Arabidopsis *Actin2* (*AtActin2*) as the endogenous reference for normalization. Expression levels were calculated by the comparative cycle threshold method, and normalization to the control was performed as described previously [67]. Three technical replicates of the qRT-PCR assay were used for each sample with a minimum of two biological replicates. Primer sequences are shown in Table S6.

### Arabidopsis PPI database

The Arabidopsis PPI dataset (∼96,221 PPIs as of AtPIN-release 8) was obtained from the *A. thaliana* protein interaction network (AtPIN; http://bioinfo.esalq.usp.br/atpin/atpin.pl). The AtPIN includes the public databases of the *A. thaliana* Protein Interactome Database (AtPID), the Predicted Interactome for *Arabidopsis*, and Arabidopsis protein–protein interaction data curated from the literature by TAIR curators, BIOGRID, and IntAct. Information obtained from AtPIN includes experimentally identified and computationally predicted protein interactions in *Arabidopsis*. We used Cytoscape 2.8.3 (http://cytoscape.org) to visualize the PPI network obtained from the AtPIN network [68]. The open source software platform, Cytoscape, was used to visualize molecular interaction networks and integrate gene expression profiles. Data were integrated with the network using attributes to map nodes or edges to specific data values of gene coexpression levels or protein functions [68]. Nodes in the network correspond to genes/proteins and the edges/lines between the nodes represent the interaction between these nodes. The shape and width of the edges indicate coexpression interaction or PPI on the exported network (Figure S2).

The network was modified to improve clarity by editing, resizing, and coloring the common up-regulated and down-regulated genes and the first interacting nodes/genes, using the Cytoscape plugin Vizmapper [69], [70]. Using the graphical properties of the selected nodes, the node size value was recolored accordingly. Common up-regulated and down-regulated genes were colored yellow and red, respectively (Figure S2). The network was further analyzed using the Cytoscape plugin, Network Analyzer [71]. The Network Analyzer results showed the attributes of the nodes and edges in the corresponding network. The results showed nodal and edge attributes such as Centrality measures, Clustering Coefficient, Topological Coefficient (TC), Number of Directed and Undirected edges, and Number of self-loops present in the network (Table S4). Based on these results, the network was then simplified by removing the nodes with a TC value of zero (that is, nodes/genes that are not a part of the coregulated network, and are considered as single interacting genes). The range of the TC values was from 0 to 1. Except for our genes of interest (*NHX2* and *EXO*), nodes with dangling edges (i.e. only one edge, and no second neighbor) were deleted from the network.

## Supporting Information

Figure S1 **Functional classes of drought- and oxidative stress-regulated genes.** Genes up-regulated by (a) drought and (c) oxidative stress; and genes down-regulated by (b) drought and (d) oxidative stress at 24 hpt compared with 0 hpt in wild-type. Gene identifications for 251 and 302 drought- and oxidative stress-up-regulated and 288 and 247 drought- and oxidative stress-down-regulated genes, respectively, were entered for this analysis. Error bars are SD. GO categories significantly over- or under-represented at *p*<0.05 are shown in black. Normalized frequency of genes to number of genes on the microarray chip was determined as described elsewhere [72].
(PDF)

Figure S2 **Co-expression network of common** ***B. cinerea*** **- and abiotic stress-regulated genes.** Nodes of commonly up-regulated genes (yellow boxes) and down-regulated genes (red boxes) by *B. cinerea*, cold, drought, and oxidative stresses. Nodes of coexpressed neighboring genes are shown in gray circles. Blue lines are edges that have direct interaction with the common regulated gene; black lines are the interaction between neighboring genes. Edges starting and ending at the same node represent homodimerization of proteins “self-loops”. Experimental and predicted interactions can be found in Table S4.
(PDF)

Table S1 **Expression levels and -fold induction of** ***BUG*** **s (a) and** ***BDG*** **s (b) in wild-type samples.**
(XLSX)

Table S2 **Expression levels and -fold induction of genes up-regulated by (a) cold-, (c) drought-, and (e) oxidative stress.** Expression levels and extent of repression of genes down-regulated by (b) cold-, (d) drought-, and (f) oxidative stress. Values were obtained from wild-type samples.
(XLSX)

Table S3 **List of probe sets/array elements and locus identifiers corresponding to genes induced by** ***B. cinerea*** **inoculation and (a) cold, (b) drought, or (c) oxidative stress; or to genes repressed by** ***B. cinerea*** **inoculation and (d) cold, (e) drought, or (f) oxidative stress.**
(XLSX)

Table S4 **Coexpression and PPI network of commonly regulated candidate genes and neighboring genes/nodes/proteins of (a) experimentally tested or computationally predicted; and (b) comprehensive set of topological parameters.**
(XLSX)

Table S5 **Regulation of genes by PPA_1_ or OPDA treatment and abiotic stress.**
(PDF)

Table S6 **List of qRT-PCR primers (sequence 5′ to 3′) used in this study.**
(PDF)
