# Supplementary figures and images for: Transcriptome Analysis Reveals Genes Commonly Induced by Botrytis cinerea Infection, Cold, Drought and Oxidative Stresses in Arabidopsis
Source: PLoS One. 2014 Nov 25;9(11):e113718. doi: 10.1371/journal.pone.0113718 (PMC4244146; doi:10.1371/journal.pone.0113718)

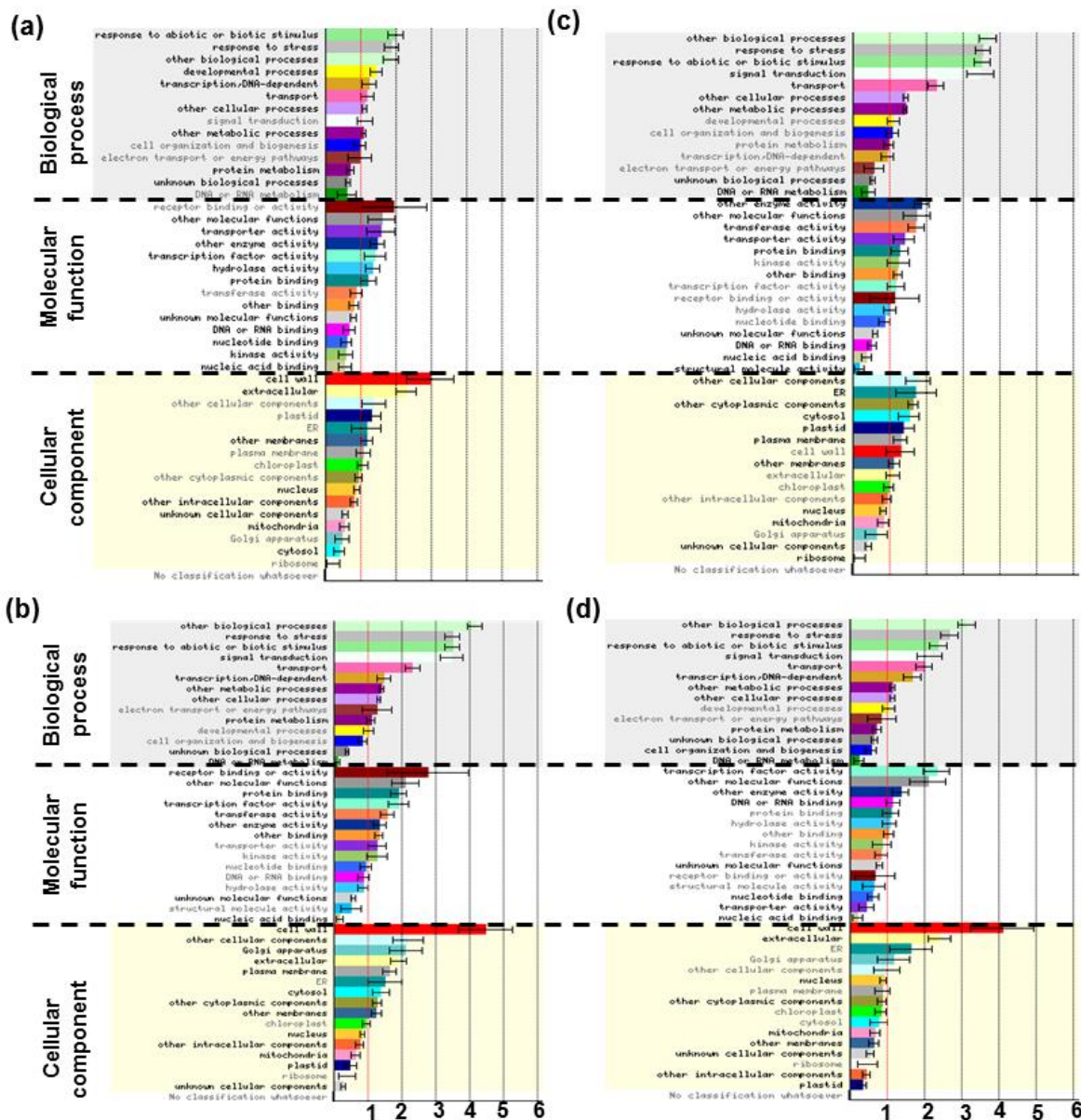

Supplement: Figure S1 — Functional classes of drought- and oxidative stress-regulated genes. Genes up-regulated by (a) drought and (c) oxidative stress; and genes down-regulated by (b) drought and (d) oxidative stress at 24 hpt compared with 0 hpt in wild-type. Gene identifications for 251 and 302 drought- and oxidative stress-up-regulated and 288 and 247 drought- and oxidative stress-down-regulated genes, respectively, were entered for this analysis. Error bars are SD. GO categories significantly over- or under-represented at p<0.05 are shown in black. Normalized frequency of genes to number of genes on the microarray chip was determined as described elsewhere [72]. (PDF) [file pone.0113718.s001.pdf]

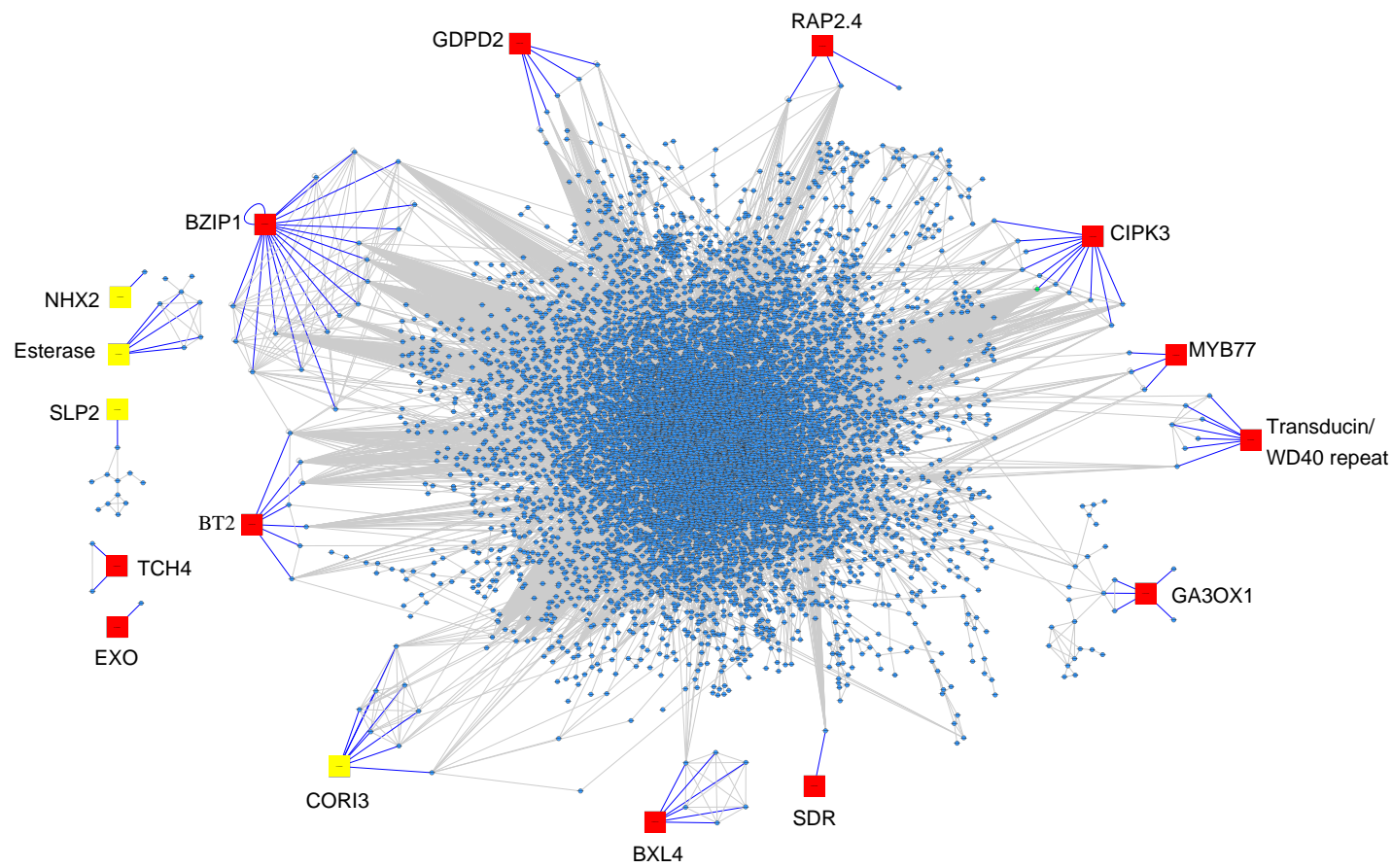

Supplement: Figure S2 — Co-expression network of common B. cinerea - and abiotic stress-regulated genes. Nodes of commonly up-regulated genes (yellow boxes) and down-regulated genes (red boxes) by B. cinerea, cold, drought, and oxidative stresses. Nodes of coexpressed neighboring genes are shown in gray circles. Blue lines are edges that have direct interaction with the common regulated gene; black lines are the interaction between neighboring genes. Edges starting and ending at the same node represent homodimerization of proteins “self-loops”. Experimental and predicted interactions can be found in Table S4. (PDF) [file pone.0113718.s002.pdf]
