# Supplementary material for: Transcriptome Analysis Reveals Genes Commonly Induced by Botrytis cinerea Infection, Cold, Drought and Oxidative Stresses in Arabidopsis
Source: PLoS One. 2014 Nov 25;9(11):e113718. doi: 10.1371/journal.pone.0113718 (PMC4244146; doi:10.1371/journal.pone.0113718)
Supplement: Table S5 — Regulation of genes by PPA1 or OPDA treatment and abiotic stress. (PDF) [file pone.0113718.s007.pdf]

Table S5. Regulation of genes by PPA_1 and/or OPDA treatment and abiotic stress.

Description	Gene locus	Normalized fold induction*		
		PPA_1§	OPDA§	Abiotic stress‡
C2H2-type zinc-finger protein related (FZF)	*At2g24500*	N	3.1	C
17.6-kD heat-shock protein (AA 1-156)	*At1g53540*	N	13.5	Ox
Class II heat-shock protein	*At5g12020*	N	12.5	Ox
Heat-shock protein 17.6A (AT-HSP17.6A)	*At5g12030*	N	13.2	Ox
Heat-shock protein family	*At5g37670*	N	3.0	Ox
Mitochondrion-localized small heat-shock protein	*At4g25200*	N	2.2	Ox
Cytochrome P450, putative (CYP72A15)	*At3g14690*	N	4.0	C
Glycosyl hydrolase family 1	*At2g44460*	N	6.1	Ox
Ser/Thr kinase-like protein	*At4g23190*	N	-3.3	D
Copper/zinc superoxide dismutase (CSD2)	At2g28190	N	-2.5	C,D,Ox
Copper Chaperine for SOD1 (CCS)	At1g12520	N	-2.5	C
Cytochrome P450, putative	*At3g14690*	11.1	N	C
Glutathione *S*-transferase (GSTU24)	*At1g17170*	61.7	N	Ox
Class I small heat shock (HSP17.6)	*At2g29500*	57.8	N	Ox
TOLB protein-related	*At4g01870*	20.1	N	Ox
β-Ig-H3 domain–containing protein/fasciclin domain–containing protein	At3g11700	-5.1	N	C
Tubulin β-8 chain (TUBB8)	At5g23860	-3.8	N	C
Fasciclin-like arabinogalactan protein (FLA2)	At4g12730	-5.1	N	C
Endo-xyloglucan transferase (TCH4)	*At5g57560*	-5.1	N	C,D
glycoside hydrolase family 28/polygalacturonase (pectinase) family	*At3g06770*	-4.1	N	C
ELI3-1	At4g37980	2.2	2.7	D

*Normalized fold induction = normalized phytoprostane-A_1 (PPA_1) or 12-oxo-phytodienoic acid (OPDA) treatment and abiotic stress/normalized no PPA_1 or OPDA treatment and no abiotic stress. Threshold value for induction/repression was at least twofold in *Arabidopsis* wild-type plants relative to controls. Fold induction by PPA_1 and OPDA (75 μM) of at least twofold in Arabidopsis plants relative to control but no induction in *tga2/5/6* at 4 hpt [32].

§OPDA or PPA_1-upregulated genes data were obtained from [47] at 3 hpt or Mueller et al. (2008) at 4 hpt, respectively.

‡Cold (C), drought (D) or oxidative stress (Ox)-upregulated genes data were obtained from this study at 24 hpt.

N, not expressed; -, downregulation.
