# Supplementary material for: Transcriptome Analysis Reveals Genes Commonly Induced by Botrytis cinerea Infection, Cold, Drought and Oxidative Stresses in Arabidopsis
Source: PLoS One. 2014 Nov 25;9(11):e113718. doi: 10.1371/journal.pone.0113718 (PMC4244146; doi:10.1371/journal.pone.0113718)
Supplement: Table S6 — List of qRT-PCR primers (sequence 5′ to 3′) used in this study. (PDF) [file pone.0113718.s008.pdf]

Table S6. List of primers (Sequence 5' to 3') used in this study.

Description	Left primer sequence	Right primer sequence
AtActin2	GTCGTACAACCGGTATTGTGCTG	CCTCTCTCTGTAAGGATCTTCATGAG
At1g73480	CTTTTCCTCCTCCTTCCGTTTCG	GGAGACCAAACCTTCCTCTCTTG
CORI3	AGATAAACAATAACCCTCCGACAGT	CTTTCAGAAAACTCTGCCTCTTATC
RD20	ATCCTTGGGAGACTTATAAGGGATT	GTAACGTAGCTGAACGCTAAGTTTATG
At2g39420	TGTATGAAGTTGCATCTAGTTCGGA	AACAGTCTCGATATTCTCTGGTGTC
EXO	CTTCATTACCTCACTCACACACACTT	GCGAGTTTGTAGTATTTTTCTGTGG
DREB26	CTTTGATGGGATCTTTTGTGGACAA	GCTCCATTATCAAACAAGAACATCC
GA4	AAGATATCACCTGTACCGAAGCTG	GAAGTGAGTTGCTTTTGTTCGAAGA
DJC24	CAAGAGATCAAATCAGCTTACCGG	GTGGATCTTCATGAAATCGTCCG
At2g20670	CTCTAGACACCTAAGAGATGTCGC	TCTATAAATTCGTGTTCCCCTGCAG
DREB2A	AGAGTGGAGATAGAAACAGAACACA	TCCATCTCTTTAATCTCTCAGCCAC
PMZ	GCAAATATTGTGGAGTCAAGTTCTG	AACTCAAAGCTTCCATAAACCTCTC
RHL41	TTGAAGAAATCTAGCAGTGGGAAGA	ATAAACTGTTCTTCCAAGCTCCAAC
REF	TTGGTTATCTTCCGTTGGTTCCTGT	CTTTCTTTCCAGCCGTATCCCCTCC
BAP1	CCCAACGAATGATTTCATGGGAAGG	TGACGATCCCACACTTATCACCAAA
UGT73B5	TTAAAGAGAGGACAACAGGGAAAGG	AATGAGTCACAAATCCTCCAATTGC
HSP17.4	GGAAGTAAAGGCGAGTATGGAGAAT	TTAACCAGAGATATCAACGGACTTG
GPX6	GTTGACAAAGATGGAAATGTTGTCG	TAAGCAGTAACTCCCAACAACTTCT
At5g35735	ACCATCATCCTCTCTATTGTCAACA	CCAAGAAAGATGAGGATCCCAATGT
At1g60730	AATATGGAATCAGGTATGCAGAGGG	GGCAACATCTACTCGCATTAAACTA
GSTU25	GTAATCCGGTATGTGAATCACTCAT	GAGCTCTTTGGTAAGGATCAGAAG
GST22/GSTU4	AAGTTCAAGTGAGAGAAAGAGAGGTC	GCCATCTCAACTCTACGAGTAAAAG
MDR4	ACGCTCTTTCTTGTAGTCTTTTGTAGC	ATATTGAGAACTTGTCCTCCTGTGTAG
ELI3-2	GGAAGTATGATAGGAGGGATAAAAGAG	CATAATCGGCAGAGATAAGCTCAAT
PDR12	GTTTCTTGAGTTTCCAGAGGAGTTTC	CCAAGCGAGTCCTAGTATGAGAAGAAA
PAD3	AACTTGTGTGTCAAGAAACTCTCTG	CGATACGACACACTATATTTCCGACTA
CYP710A1	TTGAACCACCTCGTACTCTTCATTG	TATAGTAGGGCAGTACACGATCTCA
At5g03490	TGTTATTGTTGCCGGGAACTAAATC	AAGTCAAGTAGAGGAAGTAAGTGGC
ACA12	CTCTTTGGCTCTAACACCTACCATAAG	AGACCAACAAGATCAAGATGGTTAG
At1g72900	TCAGGGTAACTACTTTGAAAGCCA	AGCAGAACCTTTTGCTTCTTGAGA
SGP2	CGAATCAACAATCTAAGGAACAGAG	CCAGGAGTACAAGCAACGATTCTA
At5g22860	GAGAAGAATCGTCGTTAGACTCTGAT	AATACCTATGCTCTATGTAGACGAGGA
RD2	AGTACAGTTTCAGGGAAGTAGTGTTG	ACATCTCTTCCTCTTCTCCTCTCTC

At5g65300	ACAGAGGAGTTTGTCCTTGTTGTTT	GGATGAAGAAGAAGAAGATCTGTGA
